# Validation and cultural adaptation of the Japanese version of the Self-Care Inventory across different research settings: a cross-sectional study

**DOI:** 10.1265/ehpm.25-00209

**Published:** 2025-10-29

**Authors:** Atsushi Takayama, Shiho Koizumi, Yoshihito Kato, Tatsuya Isomura, Tatsuyuki Hosoya, Koji Kawakami

**Affiliations:** 1 Department of Pharmacoepidemiology, Graduate School of Medicine and Public Health, Kyoto University, Japan; 2 Japan Selfcare Promotion Association; 3 Kowa Company, Ltd, Nagoya, Japan; 4 Clinical Study Support, Inc, Nagoya, Japan; 5 Department of Public Health, Nagoya City University Graduate School of Medical Sciences, Nagoya, Japan; 6 Fukuoka Prefecture Medical Center

**Keywords:** Population surveillance, Patient reported outcome measures, Self-care, Validation study

## Abstract

**Background:**

Self-care is increasingly recognized as the foundation of person-centered healthcare and a key driver for simultaneously improving population health outcomes and reducing healthcare expenditures. While the Self-Care Inventory (SCI) has been validated in several languages, Japan lacks a standardized instrument for assessing self-care in the general adult population. Moreover, it remains unclear whether the SCI reflects culturally specific self-care behaviors and retains its psychological measurement properties in non-Western contexts. Addressing both aspects, this study aimed to evaluate the Japanese version of the SCI (JSCI) in terms of its psychometric properties and its association with concrete health behaviors.

**Methods:**

We adapted the JSCI following COSMIN guidelines using forward/backward translation, expert review, and cognitive debriefing. Psychometric evaluation was based on two samples: a nationwide web-based survey (n = 504) and a community-based paper survey (n = 75). Structural validity was examined via CFA; internal consistency via Cronbach’s alpha and McDonald’s omega; and test–retest reliability via ICCs. Convergent and criterion validity were assessed through correlations with relevant psychological constructs. Measurement invariance and DIF across modes were tested, and associations with five external self-care behaviors were evaluated using AUC.

**Results:**

The hypothesized three-factor structure of the JSCI was supported across both administration modes (CFI = 0.926–0.942; SRMR < 0.06), although some subscales had elevated RMSEA. Internal consistency was acceptable to high (α = 0.75–0.85; ω = 0.81–0.92). ICCs indicated moderate to good temporal stability. JSCI scores correlated with self-care efficacy and other related constructs, supporting convergent and criterion validity. Configural invariance was confirmed, and no significant DIF was detected across modes. JSCI scores modestly discriminated individuals engaging in concrete self-care behaviors such as physical activity, strength training, Helicobacter pylori testing, and having a regular primary or dental care provider (AUCs = 0.62–0.80).

**Conclusions:**

The JSCI demonstrated satisfactory psychometric properties and structural validity across diverse research settings. Its observed associations with a range of meaningful self-care behaviors support the scale’s ecological and practical relevance in the Japanese context. The JSCI may serve as a reliable tool for evaluating and promoting self-care in both research and population health initiatives.

**Supplementary information:**

The online version contains supplementary material available at https://doi.org/10.1265/ehpm.25-00209.

## Graphical Abstract

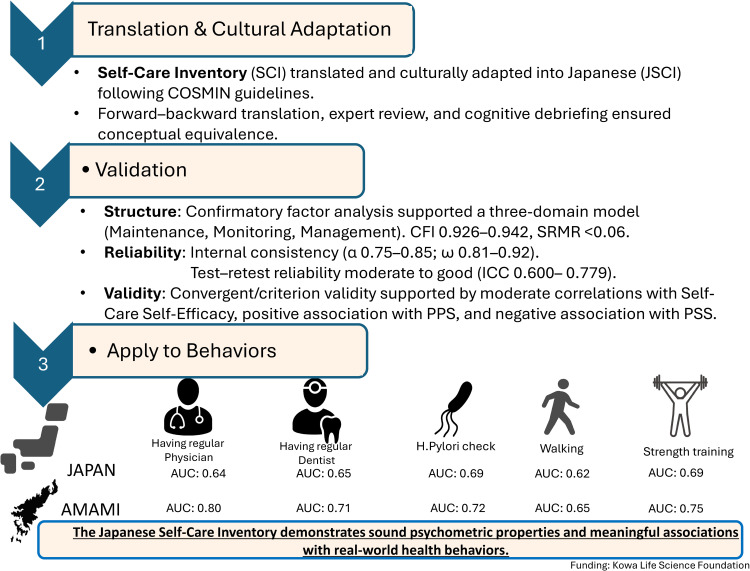

## Background

Establishing person-centered care is a growing global priority, and self-care is widely recognized as its foundational element [1]. While contemporary healthcare increasingly values patient autonomy and engagement, much of the medical literature continues to focus primarily on provider-delivered interventions rather than actions taken by individuals themselves [2–7]. This imbalance may limit our understanding of the substantial impact that everyday health behaviors have on population-level outcomes.

A substantial body of evidence has demonstrated that self-care not only improves health outcomes but also helps reduce healthcare expenditures [8–10]. These benefits are particularly relevant in countries like Japan, where rising medical demand and fiscal constraints underscore the urgency of promoting preventive, person-led health practices [11]. Despite this, Japan lacks a standardized instrument for assessing self-care in the general adult population, impeding efforts to evaluate or enhance self-care engagement on a national scale.

The Self-Care Inventory (SCI), originally developed and validated in English and Italian [3, 12], is grounded in the Middle-Range Theory of Self-Care [13] and has shown promise as a comprehensive measure of self-care maintenance, monitoring, and management [12, 13]. However, it remains unknown whether the SCI meaningfully captures culturally distinct health behaviors in non-Western contexts such as Japan.

The present study aimed to translate and culturally adapt the SCI into Japanese (JSCI), evaluate its psychometric properties, and examine whether the instrument is associated with specific, well-established self-care behaviors within the Japanese population.

## Methods

### Study design and settings

This cross-sectional study was conducted in three phases: (1) translation and cultural adaptation of the Japanese version of the Self-Care Inventory (JSCI), (2) psychometric evaluation using a web-based survey, and (3) field testing in a community-based sample. The study was conducted in accordance with the Declaration of Helsinki and Japan’s Ethical Guidelines for Medical and Health Research Involving Human Subjects. The validation process adhered to the COSMIN (Consensus-based Standards for the Selection of Health Measurement Instruments) guidelines for studies on the measurement properties of patient-reported outcome measures [14].

#### (1) Translation and cultural adaptation of the JSCI

The translation and adaptation process followed a structured methodology consistent with COSMIN guidelines. Two native Japanese speakers (AT and SK), both formally trained in English, independently translated the original English SCI into Japanese. Discrepancies were resolved through discussion, and the reconciled version was reviewed by an expert panel comprising a nurse, physician, pharmacist, and laypeople. The panel assessed content relevance, clarity, comprehensiveness, linguistic naturalness, and overall readability. Cognitive debriefing interviews with five adult volunteers led to minor wording adjustments. Two independent professional translators then back-translated the revised Japanese version into English. Discrepancies were discussed among the translators and original forward translators to ensure semantic equivalence with the original SCI. The final version was confirmed and approved by the original developer, Michela Luciani. This process was conducted between October 31, 2023, and January 24, 2024.

#### (2) Psychometric evaluation

To evaluate the psychometric properties of the JSCI, we conducted two sequential internet-based surveys using a national panel of 13 million users provided by IBRIDGE Corporation. The surveys were administered between March 6 and March 19, 2025.

In the first survey, we recruited 504 participants aged 18 years and older, stratified by age and sex to approximate the 2023 national population distribution in Japan. A subsample of 200 participants from the first survey was invited to complete the SCI again two weeks later to assess test-retest reliability.

Participant characteristics were summarized using frequencies and proportions for categorical variables, and medians with interquartile ranges (IQRs) or means with standard deviations (SD) for continuous variables. We computed item-level descriptive statistics for all 20 items of the Japanese Self-Care Inventory (JSCI), including mean, standard deviation (SD), item difficulty, item discrimination, and Cronbach’s alpha if the item was deleted. These analyses were conducted using responses from Survey 1. Structural validity was examined via confirmatory factor analysis (CFA), assuming a two-factor structure for the Self-Care Maintenance and Management scales and a one-factor structure for Monitoring, using the robust maximum likelihood estimator in accordance with the original study [12]. For the Management domain, based on theoretical justification and consistent with the original English version of the SCI, we allowed a correlated residual between Item 15 (“Change diet”) and Item 16 (“Change activity”). This adjustment was made to improve model fit while maintaining conceptual consistency [12]. Model fit was assessed using comparative fit index (CFI) (>0.95), Tucker-Levis Index (TLI) (>0.90), root mean square error of approximation (RMSEA) (<0.06), standardized root mean square residual (SRMR) (<0.08) [15]. Based on prior derivation [12] and validation studies [3], Item #14 was excluded from the CFA model. Internal consistency was evaluated using Cronbach’s α and McDonald’s ω (threshold >0.70). While alpha assumes essential tau-equivalence across items, omega allows for varying factor loadings and is therefore considered a more accurate estimate of reliability when this assumption is violated. Given this, omega values were emphasized in interpreting internal consistency. For multidimensional scales, composite reliability and the global reliability index were calculated [16, 17]. Factor score determinacy (threshold ≥0.70) was also assessed [18]. Test-retest reliability was evaluated using the intraclass correlation coefficient (ICC), with values ≥0.70 considered acceptable [18, 19]. In accordance with the validation method used for the original SCI [12], criterion validity was tested via Pearson correlation between the SCI and the Self-Care Efficacy Scale [20]. Construct validity was evaluated by testing theoretical hypotheses: higher positivity using the Prioritizing positivity scale (PPS) [21, 22] and lower perceived stress using Perceived stress scale (PSS) [23] would correlate with higher SCI scores. Correlation strength was interpreted as weak (0.10–0.29), moderate (0.30–0.49), or strong (≥0.50) [24].

#### (3) Association between JSCI and self-care behaviors related the 7 piler of selfcare component

(field testing of the SCI in a community-based setting)

To ensure that the JSCI measured the same underlying constructs across administration modes, we first conducted a multi-group confirmatory factor analysis (MG-CFA) comparing web-based and paper-based responses. Only configural invariance was assessed, and the hypothesized three-factor structure was supported in both groups, suggesting conceptual equivalence of the measure across modes. In parallel, we conducted differential item functioning (DIF) analysis using multiple-group Item Response Theory (IRT) models to evaluate potential item-level bias between modes. Based on this structural and item-level equivalence assessment, we further explored the complementary validity of the JSCI by examining its association with real-world self-care behaviors that are conceptually related to, but not directly assessed by, the JSCI. These behaviors reflect key elements of the seven pillars of self-care [25, 26], and their associations with JSCI scores were assessed to support the broader applicability and content relevance of the instrument in the Japanese context. This analysis was conducted using two datasets: the aforementioned nationwide web-based survey and a community-based paper survey conducted in Setouchi Town, Amami Oshima. In the latter, we recruited 75 local residents who participated in a health event organized by the local government on March 3, 2024. In both datasets, we applied decision tree models to examine the associations between the three JSCI factor scores—Self-Care Maintenance, Monitoring, and Management—and five binary health behavior outcomes: having a regular primary care physician [27], having a regular dental care provider [28], having ever undergone Helicobacter pylori testing [29], walking more than 8,000 steps or for over an hour at least once per week [30], and performing strength training ≥1 day per week [31]. We selected decision tree models due to their robustness in small-sample settings and ability to capture complex, non-linear relationships without requiring strict parametric assumptions [32]. Logistic regression was not employed given the limited number of events per variable, which may compromise estimate stability [33]. The discriminative ability of the JSCI scores for each behavior was evaluated using the area under the receiver operating characteristic curve (AUC). We also assessed the Pearson correlations between the JSCI’s three factor scores and other health-related constructs, including health literacy (HLS-12) [34], psychological distress (K6) [35], loneliness (Three-Item Loneliness Scale) [36], and health-related quality of life (EQ-5D-5L) [37]. All analyses were conducted using R version 4.4.2 (2024-10-31).

## Results

### Participant characteristics

Table 1 summarizes the characteristics of participants in the two distinct phases of the study: Survey 1 (n = 504), a nationwide web-based survey, and Survey 2 (n = 75), a field-based paper survey in a rural community setting. These two samples were designed for different methodological purposes and were not intended to be directly comparable. In the survey 1, participants were recruited to approximate the national distribution by age and sex, whereas the survey 2 involved older, predominantly female individuals attending a community health event. Reflecting the different sampling frames, median age was higher in Survey 2 (67 vs. 54 years), and female participation was more prominent (85% vs. 52%). Sociodemographic factors such as marital status, education level, and household income distribution also varied. Chronic condition prevalence (e.g., hypertension, dyslipidemia) was higher in the survey 2, while health behavior indicators (e.g., smoking, alcohol consumption) and psychosocial measures (e.g., Kessler-6, loneliness scale) suggested lower psychological distress but more limited health literacy in both groups. Despite these contextual differences, mean scores on the three subscales of the JSCI—Self-Care Maintenance, Monitoring, and Management—were numerically higher in the field-based sample, supporting its potential relevance in diverse settings.

**Table 1:** Participants’ characteristics

**Characteristic**	**N = 504** **(Survey 1)**	**N = 75** **(Survey 2)**
Sex -no, (%)		
Female	261 (52%)	64 (85%)
Male	243 (48%)	11 (15%)
Age (years)	54 (40, 70)	67 (50, 76)
Height (cm)	162 (156, 170)	154 (148, 160)
Weight (kg)	57 (49, 65)	54 (49, 62)
Married -no, (%)	276 (55%)	66 (88%)
Living with child(ren) ≤18 years -no, (%)	254 (50%)	11 (15%)
Household Size -no, (%)		
1 (living alone)	97 (19%)	1 (1.4%)
2	185 (37%)	22 (30%)
3	125 (25%)	31 (42%)
4	67 (13%)	8 (11%)
5	30 (6.0%)	10 (14%)
6	0	2 (2.7%)
Highest education level -no, (%)		
Elementary school	2 (0.4%)	
Junior high school	12 (2.4%)	10 (14%)
High school	165 (33%)	25 (34%)
Junior college	49 (9.7%)	10 (14%)
Professional training college	215 (43%)	15 (20%)
Technical school	36 (7.1%)	4 (5.4%)
University	22 (4.4%)	10 (14%)
Graduate school	3 (0.6%)	
Caregiver (yes) -no, (%)	39 (7.7%)	11 (15%)
Annual household income -no, (%)		
1. <¥1 million (∼<$6.7k)	43 (8.7%)	9 (13%)
2. ¥1–2 million (∼$6.7–13.3k)	38 (7.7%)	16 (22%)
3. ¥2–3 million (∼$13.3–20k)	62 (13%)	11 (15%)
4. ¥3–4 million (∼$20–26.7k)	87 (18%)	12 (17%)
5. ¥4–5 million (∼$26.7–33.3k)	50 (10%)	5 (6.9%)
6. ¥5–6 million (∼$33.3–40k)	52 (10%)	
7. ¥6–7 million (∼$40–46.7k)	32 (6.5%)	¥5–7.5 million (∼$33.3–50K) 14 (19%)
8. ¥7–8 million (∼$46.7–53.3k)	35 (7.1%)	
9. ¥8–9 million (∼$53.3–60k)	22 (4.4%)	¥7.5–10 million (∼$50–66.7K) 3 (4.2%)
10. ¥9–10 million (∼$60–66.7k)	23 (4.6%)	
11. ¥10–12 million (∼$66.7–80k)	27 (5.4%)	2 (2.8%)
12. ¥12–15 million (∼$80–100k)	13 (2.6%)	0 (0%)
13. ¥15–18 million (∼$100–120k)	8 (1.6%)	0 (0%)
14. ¥18–20 million (∼$120–133.3k)	4 (0.8%)	0 (0%)
15. >¥20 million (∼>$133.3k)	0 (0%)	0 (0%)
Smoking status -no, (%)		
Current	97 (19%)	3 (4.1%)
Never	297 (59%)	50 (68%)
Past	110 (22%)	21 (28%)
Alcohol consumption frequency -no, (%)		
<1/week	98 (19%)	10 (14%)
1–2 days/week	32 (6.3%)	10 (14%)
3–4 days/week	26 (5.2%)	6 (8.1%)
5–6 days/week	64 (13%)	3 (4.1%)
Never	284 (56%)	6 (8.1%)
Hypertension -no, (%)	117 (23%)	29 (39%)
Diabetes -no, (%)	23 (4.6%)	6 (8.1%)
Dyslipidemia -no, (%)	66 (13%)	20 (27%)
Periodontal disease -no, (%)	89 (18%)	16 (22%)
Malignancy -no, (%)	27 (5.4%)	5 (6.7%)
Stroke -no, (%)	15 (3.0%)	0
Myocardial infarction -no, (%)	13 (2.6%)	5 (6.7%)
Heart failure -no, (%)	5 (1.0%)	1 (1.3%)
Chronic kidney disease -no, (%)	11 (2.2%)	0
Pulmonary disease -no, (%)	23 (4.6%)	4 (5.3%)
Depression -no, (%)	36 (7.1%)	1 (1.3%)
Liver disease -no, (%)	13 (2.6%)	0
Rheumatic disease -no, (%)	9 (1.8%)	2 (2.7%)
Dementia -no, (%)	3 (0.6%)	0
UCLA loneliness scale ≥6 -no, (%)	180 (36%)	9 (12%)
Kessler 6 scale score -no, (%)		
0–4	302 (60%)	53 (75%)
5–9	90 (18%)	12 (17%)
10–12	41 (8.1%)	5 (7.0%)
≥13	71 (14%)	1 (1.4%)
Perceived stress scale-4	8.0 (5.0, 8.0)	8.00 (8.00, 9.00)
Prioritizing positivity scale	30 (24, 39)	21.0 (19.0, 24.0)
EQ-5D-5L	0.95 (0.82, 1.00)	1.00 (0.87, 1.00)
Health Literacy Survey Questionnaire 12 -no, (%)		
Low	362 (75%)	58 (77%)
Moderate	56 (12%)	10 (13%)
Intermediate	43 (8.9%)	5 (6.7%)
High	20 (4.2%)	2 (2.7%)
Self-care Maintenance scale	62 ± 18	69 ± 16
Self-care Monitoring scale	60 ± 21	65 ± 21
Self-care Managing scale	52 ± 20	56 ± 20

n (%); Median (Q1, Q3); Mean ± SD

### Result of psychometric evaluation

#### Item-level statistics and internal consistency

Descriptive statistics for each of the 20 items of the JSCI are summarized in Supplementary Table 1. All items had complete responses with no missing data. The mean item scores ranged from 2.29 to 4.02, and skewness values were generally modest, suggesting approximate symmetry. Item difficulty values ranged from 0.46 to 0.80, and item discrimination values ranged from 0.33 to 0.71. The mean inter-item correlation was 0.292. Cronbach’s alpha if an item was deleted remained stable across all items, indicating no single item unduly inflated or suppressed internal consistency.

#### Structural validity

CFA supported the hypothesized structure of the JSCI, comprising a two-factor model for the Self-Care Maintenance and Management subscales, and a one-factor model for the Monitoring subscale. Model fit indices are presented in Table 2. The CFI ranged from 0.926 to 0.942, and all SRMR values were below the 0.06 threshold, indicating acceptable fit. However, the RMSEA values for the Monitoring (0.186) and Management (0.129) subscales exceeded the conventional cutoff of 0.06, suggesting potential misspecification in these models.

**Table 2:** Model fit indices of the three subscales of the JSCI (n = 504)

**Subscales**	**Scaled χ^2^**	**df**	**CFI**	**TLI**	**RMSEA**	**SRMR**
Self-care maintenance scale	52.65	13	0.926	0.886	0.087	0.0478
Self-care monitoring scale	40.47	5	0.940	0.891	0.186	0.0508
Self-care management scale	52.95	7	0.942	0.861	0.129	0.0520

JSCI: Japanese version of self-care inventory, χ^2^: chi-square, df: degree of freedom, CFI: Comparative Fit Index, TLI: Tucker-Lewis Index, RMSEA: Root Mean Square Error of Approximation, SRMR: Standardized Root Mean Square Residual

Composite reliability was computed for each factor within the three subscales of the JSCI. For the Self-Care Maintenance subscale, CR was 0.704 for the Health Promoting factor, indicating acceptable internal consistency, and 0.568 for the Illness Related factor, suggesting suboptimal reliability. The Monitoring subscale demonstrated excellent internal consistency with a CR of 0.881. For the Management subscale, the Autonomous and Consulting factors had CRs of 0.655 and 0.676, respectively, indicating marginal reliability levels that fall slightly below the recommended threshold of 0.70. Global Reliability Index (GRI) values were also computed to assess the precision of factor score estimation. The Monitoring subscale demonstrated the highest GRI (0.620), indicating good reliability of factor scores. In contrast, the Health Promoting (0.386) and Illness Related (0.305) factors within the Maintenance subscale showed relatively low GRIs, suggesting limited reliability in estimating latent scores from observed variables. GRI values for the Autonomous (0.401) and Consulting (0.531) factors of the Management subscale were moderate, with the latter approaching the acceptable range (≥0.60). These results highlight variability in score precision across subscales and support the need for further refinement of the Illness Related and Autonomous domains.

Internal consistency was acceptable to good across all subscales. Cronbach’s alpha was 0.75 (95% CI: 0.71–0.78) for the Self-Care Maintenance subscale, 0.85 (95% CI: 0.83–0.87) for Monitoring, and 0.80 (95% CI: 0.77–0.82) for Management. The corresponding McDonald’s omega coefficients were 0.81, 0.92, and 0.89, respectively.

Test-retest reliability was assessed in a subsample of 200 participants using intraclass correlation coefficients (ICCs). The Self-Care Maintenance subscale showed good temporal stability (ICC = 0.779; 95% CI: 0.717–0.828), and the Monitoring subscale demonstrated acceptable reliability (ICC = 0.721; 95% CI: 0.647–0.781). The Management subscale yielded a lower ICC of 0.600 (95% CI: 0.503–0.682), indicating moderate test–retest reliability.

**Fig. 1:**
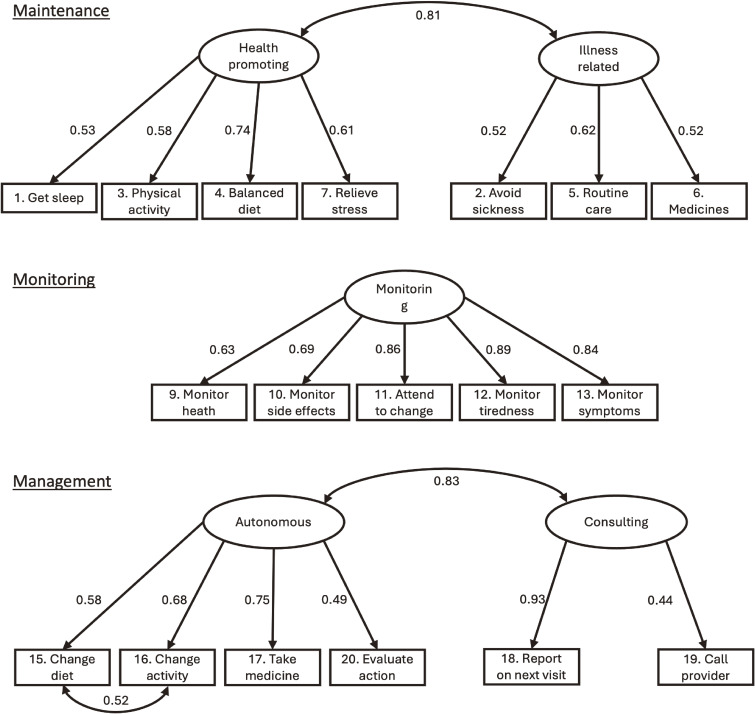
Graphical representation of the three subscales of the JSCI Standardized factor loadings from the confirmatory factor analysis of the Japanese Self-Care Inventory (JSCI). The Self-Care Maintenance and Management subscales were modeled using two-factor structures, and the Monitoring subscale followed a one-factor structure. Factor loadings ranged from 0.53 to 0.81, indicating moderate to strong associations with latent constructs

Figure 1 presents the standardized factor loadings of the final CFA model for the three subscales of the JSCI. All loadings exceeded 0.50, indicating moderate to strong associations between observed items and their respective latent constructs.

Criterion validity was supported by strong correlations with the Self-Care Efficacy Scale: Self-Care Maintenance (r = 0.540, 95% CI: 0.475–0.599), Monitoring (r = 0.540, 95% CI: 0.475–0.599), and Management (r = 0.442, 95% CI: 0.369–0.510). Construct validity was assessed by testing theoretically derived hypotheses. Positivity was moderately positively correlated with all three subscales—Maintenance (r = 0.432, 95% CI: 0.358–0.500), Monitoring (r = 0.489, 95% CI: 0.420–0.553), and Management (r = 0.382, 95% CI: 0.305–0.455). Perceived stress showed negative correlations with Maintenance (r = −0.370, 95% CI: −0.443 to −0.293), Monitoring (r = −0.254, 95% CI: −0.334 to −0.171), and Management (r = −0.171, 95% CI: −0.254 to −0.085). All correlations were consistent with the hypothesized directions.

### Result of the association between JSCI and self-care behaviors related the 7 piler of selfcare component

Configural invariance was examined using MG-CFA to assess whether the hypothesized three-factor structure of the JSCI was consistently represented across administration modes (web-based vs. paper-based). Acceptable model fit was observed for all three subscales in both groups. Specifically, the Maintenance subscale showed a CFI of 0.919 and a RMSEA of 0.091; the Monitoring subscale yielded a CFI of 0.943 and RMSEA of 0.187; and the Management subscale demonstrated a CFI of 0.952 and RMSEA of 0.118. These findings support the configural invariance of the JSCI, suggesting that the conceptual structure of the scale is preserved across administration modes, despite some variation in fit indices. DIF was also assessed using multiple-group IRT models. No statistically significant DIF was identified for any item across administration modes.

To evaluate the complementary validity of the JSCI, we examined its association with five real-world self-care behaviors not directly assessed by the scale but conceptually related to the seven pillars of self-care. In both the nationwide web-based and community-based paper surveys, JSCI scores demonstrated modest to acceptable discriminative ability in identifying individuals who engaged in these behaviors. Descriptive frequencies for these five behaviors in both samples are presented in Supplemental Table 2.

**Fig. 2:**
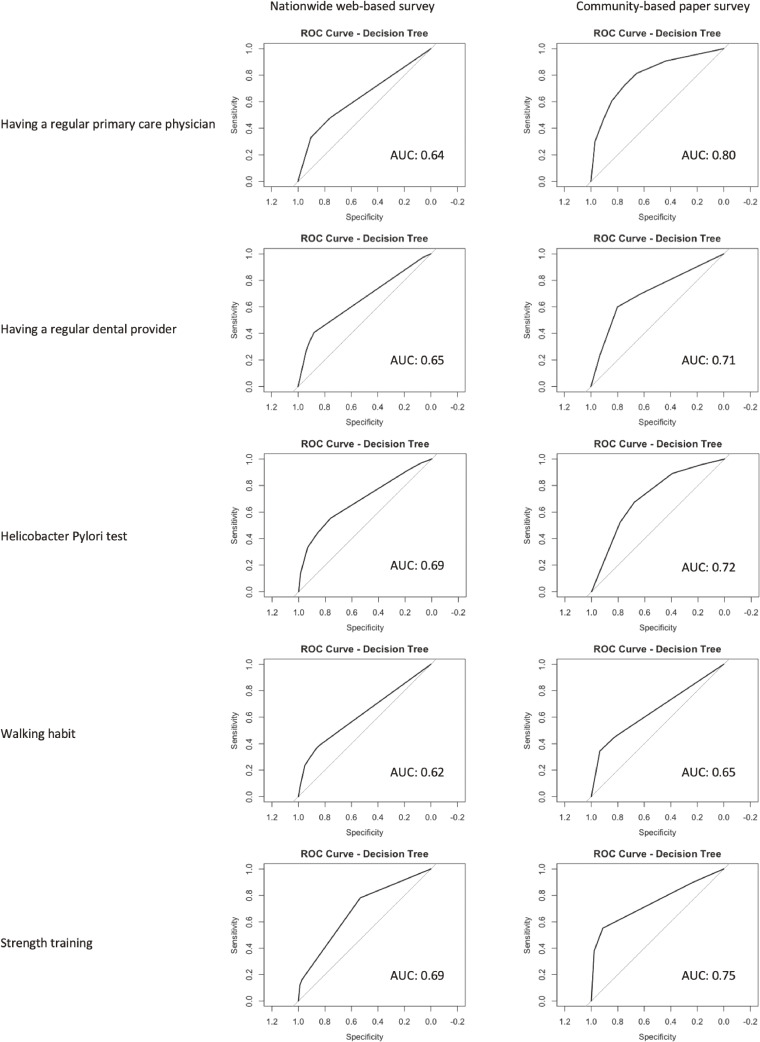
External Discriminative Validity of the JSCI Across Two Settings: AUCs for Five Self-Care Behaviors Area under the receiver operating characteristic curve (AUC) values from decision tree models incorporating all three JSCI subscale scores (Maintenance, Monitoring, Management) as predictors. Models were separately constructed in two samples: a nationwide web-based survey and a community-based paper survey conducted in Setouchi Town, Amami Oshima. Each AUC indicates the discriminative ability of the JSCI to classify participants by engagement in specific self-care behaviors, including having a regular primary care physician, having a regular dental care provider, ever undergoing Helicobacter pylori testing, walking more than 8,000 steps or over one hour at least once per week, and engaging in strength training at least once per week. *Note.* All three JSCI subscale scores (Maintenance, Monitoring, Management) were included simultaneously in each model. AUC values reflect the discriminative ability for each binary self-care behavior. Results are presented separately for the nationwide web-based survey and the community-based survey in Setouchi Town, Amami Oshima. Discrimination was interpreted based on established AUC thresholds: 0.50 = no discrimination, 0.60–0.70 = modest, 0.70–0.80 = acceptable, 0.80–0.90 = excellent, and ≥0.90 = outstanding.

In the web-based survey, the area under the ROC curve (AUC) ranged from 0.62 for walking habit to 0.69 for Helicobacter pylori testing. Notably, the AUCs were 0.64 for having a regular primary care physician, 0.65 for regular dental visits, and 0.69 for strength training at least once per week (Fig. 2).

In the community-based survey conducted in Setouchi Town, the AUCs were generally higher, ranging from 0.65 (walking habit) to 0.80 (regular primary care physician). Other outcomes included AUCs of 0.71 for regular dental care, 0.72 for Helicobacter pylori testing, and 0.75 for strength training (Fig. 2). These results suggest that JSCI scores may have practical utility in identifying individuals who engage in various health-promoting behaviors across diverse populations.

Additional construct validity was supported by correlations in the hypothesized directions. Table 3 summarizes the associations between the three SCI subscales and external measures, including PPS, PSS, HLS-12, K6, loneliness, and EQ-5D-5L. Negative correlations were found with HLS-12, K6, and loneliness, consistent with theoretical expectations. Associations with EQ-5D-5L were weak or negligible.

**Table 3:** Construct Validity: Correlations Between the SCI Subscales and Conceptually Related External Variables

	**Maintenance**	**Monitoring**	**Management**
PPS	0.43(0.36, 0.50)	0.48 (0.42, 0.55)	0.38 (0.31, 0.45)
PSS	−0.37 (−0.44, −0.29)	−0.25 (−0.33, −0.17)	−0.17 (−0.25, −0.08)
HLS-12	−0.40 (−0.47, −0.32)	−0.33 (−0.41, −0.25)	−0.21 (−0.30, −0.13)
K6	−0.28 (−0.36, −0.19)	−0.11 (−0.20, −0.03)	−0.00 (−0.09, 0.08)
Loneliness	−0.27 (−0.35, −0.19)	−0.15 (−0.23, −0.06)	−0.06 (−0.14, 0.03)
EQ-5D-5L	0.19 (0.11, 0.28)	0.07 (−0.02, 0.16)	−0.00 (−0.09, 0.09)

Notes, Values represent Pearson correlation coefficients with 95% confidence intervals shown in parentheses. SCI = Self-Care Inventory; PPS = Prioritizing positivity scale, PSS = Perceived Stress Scale; HLS-12 = 12-item Health Literacy Scale; K6 = Kessler Psychological Distress Scale; EQ-5D-5L = EuroQol 5-Dimension 5-Level.

## Discussion

### Key results

This study evaluated the measurement properties of the JSCI using both web-based and community-based samples. The instrument showed acceptable internal consistency, moderate to strong factor loadings, and a replicable factor structure across administration modes, supporting its structural validity. While composite and global reliability varied across subscales, test-retest reliability and construct validity were broadly acceptable. Criterion validity was supported by moderate to strong correlations with self-care efficacy, and hypothesized correlations with positivity and stress further supported construct validity. Moreover, the JSCI demonstrated configural invariance between administration modes and showed modest discriminative ability for identifying real-world self-care behaviors conceptually aligned with the seven pillars of self-care.

### Comparison with previous studies

Two previous studies have evaluated translated versions of the SCI: the English version by Luciani et al. [3] and the Arabic version by Rababah et al. [38]. All three studies, including ours, confirmed the original three-factor structure of the SCI. In our study, CFA demonstrated acceptable to good model fit (CFI = 0.926–0.942, RMSEA = 0.087–0.187), which is broadly consistent with values reported by Luciani et al. (0.938–0.977, RMSEA = 0.035–0.085) and Rababah et al. (CFI = 0.964–0.994, RMSEA = 0.040–0.069).

Measurement invariance was assessed using multi-group CFA and IRT-based DIF analysis. While Luciani et al. evaluated cross-cultural invariance (Italy vs. U.S.) [3] and Rababah et al. examined gender-based invariance [38], our study focused on administration mode (web vs. paper). All three studies confirmed configural invariance, and our results additionally showed no evidence of differential item functioning, supporting equivalence at both the structural and item levels.

Internal consistency was high across all studies. Cronbach’s alpha values in our study ranged from 0.75 to 0.85 across subscales, comparable to the Italian version (0.69–0.84) [3] and slightly lower than the Arabic version (0.82–0.86) [38], indicating acceptable to good internal consistency across all language versions.

With regard to convergent validity, all three studies examined the association between SCI scores and self-efficacy [3, 38]. Luciani et al. reported moderate to strong correlations between SCI subscales and self-care agency (r = 0.57–0.64) [3], while Rababah et al. found similar correlations with general self-efficacy (r = 0.56–0.68) [38]. In our study, self-efficacy was also significantly associated with each of the JSCI subscales, with correlation coefficients ranging from 0.442 to 0.540. These consistently positive associations across language versions and populations support the theoretical coherence of the SCI, as self-efficacy is a core component of self-care theory [13].

Importantly, to our knowledge, no previous studies have directly examined the relationship between SCI scores and specific, observable self-care behaviors outside of the items included in the scale itself. In contrast, our study uniquely evaluated associations between the JSCI and a set of distinct health-related behaviors that are conceptually relevant but not explicitly measured by the SCI. Although the discriminatory capacity of the JSCI was modest (AUCs ∼0.62–0.80), consistent associations across different settings (web-based and community-based) suggest that the JSCI may reflect real-world engagement in health-promoting behaviors. This supports the practical applicability of the scale in real-world settings. Taken together, the psychometric properties of the JSCI are largely consistent with previous validations in other languages, supporting its reliability and conceptual equivalence across populations and administration formats.

### Limitations

However, our study has several limitations. First, although we conducted validation across two distinct populations—a nationwide web-based sample and a community-based field sample—the generalizability of our findings remains limited. Participants in the web-based survey may have had higher digital literacy and health awareness than the general population, while the field sample was older and predominantly female. Nonetheless, the consistent factor structure observed across both samples supports the structural robustness of the JSCI across different contexts. Second, the responsiveness of the JSCI—its ability to detect changes over time or in response to interventions—was not evaluated in this study. Future longitudinal or interventional research will be necessary to establish this important measurement property. Third, although most fit indices in the CFAs were within acceptable ranges, the RMSEA values for the Monitoring and Management subscales exceeded conventional thresholds, suggesting some potential model misspecification. These findings warrant cautious interpretation and further model refinement. Fourth, while composite reliability values generally supported internal consistency, two factors in the Management subscale (Autonomous and Consulting) yielded values slightly below the commonly accepted threshold of 0.70. Similarly, the global reliability indices (GRI) for some subdomains, such as Health Promoting and Illness Related within the Maintenance subscale, were modest. These results suggest variability in score precision across subscales and indicate areas where further refinement may enhance measurement reliability.

### Implications and future directions

Despite these limitations, the JSCI exhibited acceptable psychometric properties and structural consistency across different populations, supporting its applicability in both research and clinical contexts. Notably, the Management subscale showed relatively lower reliability and score stability compared to other domains. This may reflect a design trade-off: while the items were intentionally framed to accommodate a wide range of symptoms across the general population, this breadth may have led to interpretive variability depending on each respondent’s current symptom experience. Future refinements—such as specifying a reference period or clarifying the types of symptoms—may improve the conceptual coherence and reliability of this subscale.

## Conclusions

In line with previous validation studies of the SCI, this study supports the structural validity and overall psychometric soundness of the Japanese version. The JSCI demonstrated adequate measurement properties for assessing self-care maintenance, monitoring, and management in the general adult population in Japan. While certain subscales, such as management, may benefit from further refinement, the scale appears suitable for use in both community-based research and web-based self-assessment settings, where efficient, scalable tools are increasingly needed.

## Supplementary information

**Additional file 1:** **Supplemental methods** Details of the questionnaires used in this study. **Supplementary Table 1.** Descriptive item statistics of the JSCI (N = 504). **Supplemental Table 2.** Descriptive Statistics for Five Self-Care Behaviors in Nationwide and Community-Based Samples.

## Declarations

### Availability of data and materials

The data used in this study are not publicly available but may be shared upon reasonable request. Deidentified individual participant data, along with a data dictionary and study protocol, may be made available after approval by the relevant institutional data management committees and ethics boards. Requests should be submitted to the corresponding author and will be evaluated based on the research purpose and compliance with ethical and legal considerations. Data sharing will require a data use agreement and may involve additional conditions set by the data custodian institution.
