# Supplementary material for: Validation and cultural adaptation of the Japanese version of the Self-Care Inventory across different research settings: a cross-sectional study
Source: Environ Health Prev Med. 2025 Oct 29;30:85. doi: 10.1265/ehpm.25-00209 (PMC12583971; doi:10.1265/ehpm.25-00209)
Supplement: Supplementary file 1 — Additional file 1: Supplemental methods Details of the questionnaires used in this study. Supplementary Table 1. Descriptive item statistics of the JSCI (N = 504). Supplemental Table 2. Descriptive Statistics for Five Self-Care Behaviors in Nationwide and Community-Based Samples. [file ehpm-30-085-s001.docx]

**Supplemental materials**

**Title**

Validation and cultural adaptation of the Japanese version of the Self-Care Inventory across different research settings: a cross-sectional study

**CONTENTS**

**Supplemental methods**

- Details of the questionnaires used in this study

**Supplemental tables**

- **Supplementary Table 1.** Descriptive item statistics of the JSCI (N=504)
- **Supplemental Table 2.** Descriptive Statistics for Five Self-Care Behaviors in Nationwide and Community-Based Samples

***Details of original questionnaires used in this study***

The original version of the Self-Care Inventory and the Japanese version (JSCI) are freely available at:

(<https://self-care-measures.com/available-self-care-measures-patient-versions/self-care-inventory-sci/>)

We also used Japanese version of Perceived stress scale-4 (PSS), Prioritizing Positivity Scale (PPS), UCLA 3 item loneliness scale, Kessler 6, EQ5D-5L, and Health Literacy Survey Questionnaire 12 (HLS-12).

Original questionnaire items on demographics and self-care behaviors

*Sex*

*Man / Woman*

*Age*

_____ years old

*Height*

How tall are you?

____cm

*Weight*

How much do you weigh?

_____kg

*Marital status*

Please choose your marital status from the following.

1. Never married

2. Married and living together

3. Married but not living together

4. Separated

5. Widowed

6. Others

*Living with child(ren) ≤ 18 years*

Do you currently live with any children under 18 years of age?

Yes / No

*Household Size*

How many people live in your household, including yourself?

___person(s)

*Highest Education level*

Please choose the highest education you have graduated from.

1. Elementary school

2. Junior high school

3. High school

4. Junior college

5. Professional training college

6. University

7. Graduate school

8. Others

*Caregiving*

Are you giving care to someone in your family?

1. Yes. I am the main caregiver for the person.

2. Yes. I partially support the person, not as a main caregiver.

3. No.

*Annual household income*

What was your household's annual income (including pension and before tax deduction) in the last year? Please choose from the following:

For the nationwide web-based survey

1. <¥1 million (~< $6.7k)

2. ¥1-2 million (~$6.7–13.3k)

3. ¥2–3 million (~$13.3–20k)

4. ¥3–4 million (~$20–26.7k)

5. ¥4–5 million (~$26.7–33.3k)

6. ¥5–6 million (~$33.3–40k)

7. ¥6–7 million (~$40–46.7k)

8. ¥7–8 million (~$46.7–53.3k)

9. ¥8–9 million (~$53.3–60k)

10. ¥9–10 million (~$60–66.7k)

11. ¥10–12 million (~$66.7–80k)

12. ¥12–15 million (~$80–100k)

13. ¥15–18 million (~$100–120k)

14. ¥18–20 million (~$120–133.3k)

15. > ¥20 million (~> $133.3k)

For the community-based paper survey in Amami island, Setouchi town

1. <¥1 million (~< $6.7k)

2. ¥1-2 million (~$6.7–13.3k)

3. ¥2–3 million (~$13.3–20k)

4. ¥3–4 million (~$20–26.7k)

5. ¥4–5 million (~$26.7–33.3k)

6. ¥5–7.5 million (~$33.3–50K)

7. ¥7.5–10 million (~$50–66.7K)

8. ¥10–12 million (~$66.7–80k)

9. ¥12–15 million (~$80–100k)

10. ¥15–18 million (~$100–120k)

11. ¥18–20 million (~$120–133.3k)

12. > ¥20 million (~> $133.3k)

*Smoking habits*

Do you smoke?

1. Never smoke.

2. I used to smoke.

3. I currently smoke.

*Drinking habits*

How often do you drink alcohol of more than 20g ethanol (500 ml beer, a cup of Japanese sake, or 100 ml Japanese shochu) in a week?

0. Never

1. 1 to 2 times a week

2. 3 to 4 times a week

3. 5 to 6 times a week

4. Everyday

*Past medical history*

Please choose diseases which you have been diagnosed with.

Hypertension

Diabetes

Dyslipidemia

Periodontal disease

Malignancy/Cancer (Stomach cancer, Lung cancer, Leukemia, Lymphoma, etc.)

Stroke (Cerebral infarction, Cerebral hemorrhage)

Acute myocardial infarction/ Angina pectoris

Heart failure

Kidney disease (Renal failure, nephrosis, hemodialysis, etc.)

Lung disease (Emphysema, Chronic obstructive pulmonary disease: COPD, Bronchitis, Asthma, etc.)

Depression

Liver disease (Hepatitis, Liver cirrhosis)

Rheumatic disease (Rheumatic arthritis, Systematic lupus erythematosus, vasculitis, etc.)

Dementia

***Attention check question***

Please choose “strongly agree” from the bottom of the following options.

1. strongly agree

2. agree

3. slightly agree

4. neutral

5. slightly disagree

6.disagree

7. strongly disagree

Please neglect the following question regarding electronic devices and choose “others” from the bottom of the following options.

Which electronic devices do you usually use most often?

1. laptop personal computer

2. desktop personal computer

3. television

4. smartphone

5. radio

6. others

*Having a regular primary care physician*

Do you have a regular primary care doctor whom you can consult about any health concerns?

Yes / No

*Having a regular dental provider*

In the past 12 months, have you had a dental check-up (such as for routine examination, cleaning, or tartar removal)?

Please do not include visits for treatment purposes such as cavities.

Yes / No

*Helicobacter Pylori test*

Have you ever been tested for Helicobacter pylori (H. pylori)?

Yes / No

*Walking habit*

Do you walk at least 8,000 steps or for more than one hour at least once a week?

Yes / No

*Strength training*

Do you engage in strength training at least once a week?

Yes / No

| *Item* | *Missing* | *Mean* | *SD* | *Item Difficulty* | *Item Discrimination* | *α if deleted* |
| --- | --- | --- | --- | --- | --- | --- |
| #1 | 0.00 % | 3.54 | 1.08 | 0.71 | 0.36 | 0.89 |
| #2 | 0.00 % | 4.02 | 1 | 0.80 | 0.48 | 0.88 |
| #3 | 0.00 % | 3.01 | 1.35 | 0.60 | 0.45 | 0.88 |
| #4 | 0.00 % | 3.56 | 1.07 | 0.71 | 0.56 | 0.88 |
| #5 | 0.00 % | 3.2 | 1.38 | 0.64 | 0.45 | 0.88 |
| #6 | 0.00 % | 3.88 | 1.22 | 0.78 | 0.48 | 0.88 |
| #7 | 0.00 % | 3.09 | 1.26 | 0.62 | 0.48 | 0.88 |
| #8 | 0.00 % | 3.7 | 1.45 | 0.74 | 0.33 | 0.89 |
| #9 | 0.00 % | 3.68 | 1.02 | 0.74 | 0.69 | 0.88 |
| #10 | 0.00 % | 3.4 | 1.17 | 0.68 | 0.65 | 0.88 |
| #11 | 0.00 % | 3.23 | 1.12 | 0.65 | 0.65 | 0.88 |
| #12 | 0.00 % | 3.32 | 1.1 | 0.66 | 0.65 | 0.88 |
| #13 | 0.00 % | 3.38 | 1.1 | 0.68 | 0.71 | 0.88 |
| #14 | 0.00 % | 3.08 | 1.45 | 0.51 | 0.42 | 0.89 |
| #15 | 0.00 % | 2.98 | 1.07 | 0.60 | 0.53 | 0.88 |
| #16 | 0.00 % | 3.12 | 1.06 | 0.62 | 0.53 | 0.88 |
| #17 | 0.00 % | 3.24 | 1.13 | 0.65 | 0.39 | 0.89 |
| #18 | 0.00 % | 3.21 | 1.15 | 0.64 | 0.49 | 0.88 |
| #19 | 0.00 % | 2.29 | 1.09 | 0.46 | 0.33 | 0.89 |
| #20 | 0.00 % | 3.23 | 1.43 | 0.54 | 0.53 | 0.88 |

**Notes,** Mean inter-item-correlation=0.292 · Cronbach's α=0.887 Item difficulty is the normalized average item score (mean score divided by the maximum possible score of 7), indicating how frequently the behavior is practiced. Item discrimination refers to the correlation between the item score and the total scale score, reflecting how well the item differentiates between individuals with different levels of overall self-care.

**Supplemental Table 2**. Descriptive Statistics for Five Self-Care Behaviors in Nationwide and Community-Based Samples

|  | N = 504  (Survey 1) | N = 75  (Survey 2) |
| --- | --- | --- |
| 1. Having a regular primary care physician | 163 (32%) | 43 (57%) |
| 2. Having a regular dental care provider | 206 (41%) | 30 (40%) |
| 3. Having ever undergone Helicobacter pylori testing | 132 (26%) | 46 (62%) |
| 4. Walking more than 8,000 steps or for over an hour at least once per week | 146 (29%) | 29 (39%) |
| 5. Performing strength training ≥1 day per week | 110 (22%) | 29 (39%) |

**Notes,** Values represent the number and percentage of participants who reported engaging in each of the five observed self-care behaviors in Survey 1 (nationwide web-based sample) and Survey 2 (community-based paper-based sample). These behaviors correspond to the binary outcomes used in the discriminative validity analysis shown in Figure 2. Percentages are based on the total number of respondents in each survey sample (N = 504 and N = 75, respectively).
